# Application of an Online Combination Exercise Intervention to Improve Physical and Mental Health in Obese Children: A Single Arm Longitudinal Study

**DOI:** 10.3389/fpsyg.2021.638618

**Published:** 2021-09-14

**Authors:** Meng Ding, Xiangren Yi, Peisai Yan, Daniel J. McDonough, Zan Gao, Xiaosheng Dong

**Affiliations:** ^1^College of Physical Education, Shandong Normal University, Jinan, China; ^2^Department of Sport and Health, School of Physical Education, Shandong University, Jinan, China; ^3^School of Kinesiology, University of Minnesota, Minneapolis, MN, United States

**Keywords:** childhood obesity, body fat percentage, quality of life, thinking, work attitude

## Abstract

**Introduction:** Childhood obesity has become a global public health concern in the past decade. The purpose of this study was to explore the effectiveness of an online combination exercise intervention in improving the physical and mental health of obese children.

**Methods:** This study adopted a one-group pre-test and post-test research design. A total of 28 obese children from 6 elementary schools in Heze City, Shandong Province, China, were recruited. All participants received an 8-week online combination exercise intervention and were tested at a three-month follow-up. Participants were tested and surveyed regarding their demographic, feasibility, and obesity indicators using mental health and quality of life questionnaires. The data were statistically analyzed using a one-way analysis of variance with repeated measures.

**Results:** A total of 26 obese children (Mean_age_ = 10.15 years) completed the study. The retention rate was 92.9% for the entire trial (two obese children withdrew due to their inability to exercise) and no adverse events were reported. In addition, the obese children completed 25/32 (78.1%) of the online courses. After 8 weeks of the intervention, the changes in the body fat percentage of the obese children [mean difference (MD) = –3.126, *p* < 0.001, Cohen’s *d* = –1.777], thinking dimension score (MD = 1.654, *p* < 0.001, Cohen’s *d* = 0.603), total quality of life score (MD = 6.385, *p* < 0.05, Cohen’s *d* = 0.610), and work attitude dimension score (MD = 1.346, *p* < 0.001, Cohen’s *d* = 0.744) showed significant differences compared to the baseline. However, no significant differences were identified between the post-intervention and three-month follow-up measurements (*p* > 0.05), and we assumed that the intervention effect was maintained three months after the intervention.

**Conclusion:** An online combination exercise intervention is a safe and feasible option to improve the mental health and quality of life of obese children and may have long-term health benefits.

## Introduction

The global prevalence of childhood obesity has tremendously increased in the past two decades and childhood obesity has become a public health concern in developed as well as developing countries (Ng et al., 2014). Childhood obesity has been shown to have significant negative effects on both physical and mental health, increasing the risk of hyperlipidemia, coronary heart disease, and type 2 diabetes mellitus (l’Allemand-Jander, 2010), as well as reducing children’s quality of life (Pavlova et al., 2016). Obesity in children is a global issue and thus has become a public health priority around the world (Wang and Lobstein, 2006). It has caused serious health and economic burdens on both global and national levels (Singh et al., 2008). Even more concerning is that the rapid development of China’s economy has led to an accelerated rate of transformation of the Chinese diet; a result, the population of children with overweight and obesity has increased significantly (Tanenbaum et al., 2017; Wang et al., 2019). Evidence suggests that obese youngsters are less physically active than non-obese ones (Janssen et al., 2004; Vandewater et al., 2004). It has been shown that regular participation in physical activity may serve as an effective strategy for preventing and curbing childhood obesity (Park et al., 2018; Gao, 2018), while combination exercise intervention has the greatest effect on obesity (Kelley et al., 2017). There are high levels of physical inactivity in children in China, especially in children with obesity. Recently, many studies have examined the various benefits of physical activity programs for children with obesity, such as weight loss (Thivel and Duche, 2014) and improvements in motor skills (Palmer et al., 2019; Gao et al., 2019b), mental health (Lu et al., 2012), and quality of life (Shoup et al., 2008**;** Martin-Garcia et al., 2019). However, there are several notable limitations in the existing exercise interventions conducted in China. The first limitation is that, given the high traffic delays in China, obese children are usually unable to adhere to regular exercise interventions at local fitness centers due to the constant traffic jams and/or guardian time conflicts. The second limitation is that the types of exercise intervention used are too narrow (e.g., including only simple aerobic exercise or resistance training), and the third is that face-to-face group exercise interventions may contribute to the spread of respiratory diseases (e.g., COVID-19). Fourth, although face-to-face private guidance may promote better social interaction and program sustainability for children, it will increase the financial burden on obese children’s families.

Recent research shows that obese children are more likely to experience serious mental health problems (Esposito et al., 2014), and mental health problems can lead to decreased physical activity and social problems in obese children (Warschburger, 2011). In addition, low physical activity levels can exacerbate children’s level of obesity children (Mokabane et al., 2014), creating a vicious circle. Physical activity can improve obese children’s happiness with their life (Armstrong and Wong, 2019) and promote a positive attitude (Tsang et al., 2013), meaning that they can better deal with the negative impacts and social maladjustment caused by obesity. In time, exercise interventions can encourage obese children to form long-term exercise and beneficial health habits (Nemet et al., 2005). However, more studies are needed to better understand the specific mechanisms responsible for the effect of physical activity on mental health (Rodriguez-Ayllon, 2019).

With the rapid development and accessibility of Internet technology, the application of emerging technologies for health promotion has aroused great public interest (Zeng et al., 2018) and it has become increasingly possible to utilize social media to help children with obesity exercise online. However, to the best of our knowledge, no such research study has been carried out in China. Therefore, the purpose of our study was to explore the effectiveness of using an online combination exercise intervention to promote physical and mental health in obese children. We anticipated that the remote physical exercise intervention (focusing on flexibility, aerobic exercise, and resistance training) would be an effective and feasible means to improve obese children’s health-related outcomes. This study is significant in that our findings might shed new light on the development and implementation of innovative and efficacious physical activity interventions for obese children facing health challenges.

## Materials and Methods

### Participants

Children with obesity were recruited by posting leaflets and carrying out word-of-mouth publicity efforts in 6 elementary schools in Heze City, Shandong Province, China. According to the “Research Methods in Education” guideline, if general conclusions about the school population as a whole are to be reached, the sample size needs to be at least 30 (Cohen et al., 2007). Our research team contacted interested children with obesity or their legal guardians. A total of 50 children were chosen for this study, but we only recruited 30 children (mean age = 10.15; age range, SD = 0.82) with obesity who met the inclusion and exclusion criteria for this study. The inclusion criteria were as follows. (1) Based on the classification criteria for Chinese school-aged children with overweight and obesity (Group of China Obesity Task Force, 2004), we included obese children with a BMI greater than or equal to their corresponding gender and age group “obese” cutoff point; (2) the children had to be 9–12 years old; (3) the children had to be able to exercise; (4) the children had to be willing to provide consent/assent. Participants satisfying one or more of the following criteria were excluded: (1) having non-simple obesity (e.g., vegetarians); (2) having a history of food allergies; (3) suffering from any of the following diseases: cardiopulmonary dysfunction, diabetes, kidney disease, liver disease, coronary heart disease, and other metabolic diseases; (4) being incapable of performing moderate- or higher-intensity exercise; (5) having taken hypoglycemic or lipid-lowering drugs, other anti-metabolic disease drugs, and/or high-dose nutritional supplements within the past 3 months; and (6) having a history of weight loss within the past 12 months.

### Study Design and Procedures

This study was conducted in Heze City, Shandong Province, China, from fall 2018 to fall 2019 using a one-group pre-test/post-test research design. In order to improve the level of knowledge of students and parents regarding childhood obesity, we delivered a lecture about childhood obesity in the school, with most of the parents in attendance. Then, researchers from our research team contacted children with obesity who were interested in participating or their guardians/parents from April to June of 2018. Interested students first came to the university laboratory for baseline screening. During this time, the participants and guardians who met the inclusion criteria provided written informed consent before participating in this study and related investigations and tests were conducted, including analyses of the participants’ demographic characteristics, feasibility and acceptability indicators, obesity indicators, and mental health and quality of life indicators. In order to ensure the standardization of the tests and minimize possible errors, the testers completed the training on test procedures and precautions. This study was approved by the Ethics Committee of Shandong Normal University (20180511).

We divided 30 obese children into 3 classes with 10 people in each class, and each class was guided by a trainer. The duration of the online combination exercise intervention was 8 weeks, with 40 min of online exercise instruction four times a week (i.e., a total of 32 classes). The exercise prescription designed by our research group was provided to participants free of charge after the intervention, and then a 3-month follow-up was conducted without any intervention. The trainer has explained to the parents and children how to carry out exercises, as well as some precautions that should be taken. In the process of intervention or after the intervention, the trainer contacted children and parents who had problems such as non-standard action and absence and gave guidance and suggestions. The basic content of the intervention included flexibility, aerobic, and resistance training. Each intervention included: (1) Flexibility exercises: 5 min of flexibility exercises (e.g., leg pressing, shoulder pressing, etc.). (2) Aerobic movements: 20 min of aerobic training with 6 basic movements of Latin dance (i.e., rotation, pulsation, movement, square stepping, New York movement, hand-to-hand). We take Latin dance as an aerobic part of the training program, which makes the exercise program easier to learn and more attractive to children. (3) Resistance exercises: strength, endurance, and functional training, with 10 min of resistance training each time (mainly using their own bodyweight). The first month mainly focused on endurance training and the second month focused more on strength training; the third month primarily consisted of functional training. (4) Relaxation: after the intervention, there were 5 min of relaxation exercises, including flapping the limbs and deep breathing (Thompson et al., 2013). In this study, the trainer instructed the participants to train and communicated with the participants online in real time during the intervention process, encouraging the participants to ask questions at any time throughout the intervention process. Furthermore, the trainer adjusted the exercise intensity according to each person’s specific situation and gradually increased the intensity along with the advancement of the intervention measures and monitored the exercise intensity through the Rate of Perceived Effort (RPE) (13–16). We followed the standardized protocol developed by the trainer according to the guidelines of the American College of Sports Medicine in teaching the children. During the whole intervention process, all different sessions of the training were conducted by a well-trained trainer to ensure the smooth progress of the physical activity and exercise intervention. Side effects and serious accidents were recorded by the trainer and reported to the study’s principle investigator. The principle investigator would take further measures and perhaps terminate the training if necessary. In addition, before this study, we conducted safety education for children and parents and asked parents to accompany each exercise intervention to ensure safety during the training.

### Measurements

All of the indicators in this study were investigated and tested at baseline, after 8 weeks of the intervention, and at the end of the 3-month follow-up, including demographics, feasibility indicators, BMI, waist-to-hip ratio, body fat percentage, and mental health, as well as the quality of life indicators. The same tester tested all the participants and at the University laboratory. The test was carried out in strict accordance with the precautions and operating rules required by the measurement to ensure the preciseness of the test.

#### Demographics

All children with obesity reported their date of birth, gender, grade, and guardian’s contact information.

#### Feasibility Indicators

The feasibility of the study was evaluated by: (1) the recruitment rate (e.g., the ratio of children who participated in the intervention to the number of qualified participants); (2) the project retention rate (e.g., the proportion of participants who completed the intervention); (3) adverse events (e.g., any adverse events related to the intervention); and (4) participation rate of intervention courses (e.g., the proportion of children with obesity who participated in intervention courses) (Bellamy et al., 2020).

#### Obesity Indicators

The following indicators were measured, height [meter(m); Seca 213 Stadiometer], weight [kilogram(kg); Tanita BC-1000 Digital Weight Scale], waist/hip circumference (cm; umbilicus), and the skinfold thickness of the triceps brachii, anterior superior iliac spine, and abdominal skinfolds for boys (mm) and the skinfold thickness of the triceps brachii, chest, and subscapularis for girls (mm). (1) BMI: body weight in kilograms divided by the square of height in meters (kg/m^2^); (2) waist-to-hip ratio: waist circumference/hip circumference; and (3) body fat percentage: the percentage of body fat was measured using the skinfold thickness method. The body density of boys and girls was calculated by using the three-point method of skinfold measurements, and the equation for body fat percentage was: (4.95/body density –4.5) *100 (Thompson et al., 2013).

#### Mental Health Indicators

The mental health status of the participants was assessed using the Child and Adolescent Mental Health Scale (Warschburger, 2011). The children and adolescent mental health scale was divided into 5 subscales of cognition, thinking, personality, emotions, and volitional behavior and includes 24 items. Each item is divided into 7-point likert scale and the scoring was based on 4 positive ratings of 1, 2, 3, and 4 points. The higher the score was, the better the mental health level was. The scale has been proved to have acceptable reliability and validity and met the mental health test standards (Huo et al., 2006).

#### Quality of Life

Participants’ quality of life was assessed using the Child and Adolescent Quality of Life Scale (Wu et al., 2006). The 13 dimensions of the scale were: self-satisfaction, teacher-student relationship, somatic sensation, peer relationship, parent-child relationship, athletic ability, learning ability and attitude, self-concept, negative emotions, work attitude, activity opportunity, and life convenience and other dimensions. Each statement was assessed on a 5-point Likert scale and a score for each dimension was calculated, with a higher score indicating a better quality of life. The scale has been observed to have acceptable reliability and validity and met the requirements of the quality of life measurement (Wu et al., 2006).

### Data Analysis

We employed a univariate analysis using the SPSS 25.0 statistical software (IBM Corp., Armonk, NY, United States) for descriptive statistics. Analysis of variance (ANOVA) with repeated measures was performed to assess intragroup changes between the baseline and post-intervention indicators and changes between post-intervention and 3 months follow-up. Efficacy was estimated by segmentations from pre- to post-intervention and from post-intervention to the 3 months follow-up. Within the group, we calculated the effect value and assessed the changes in the 95% confidence interval—that is, the standardized mean difference before and after the intervention—and classified the effect size according to Cohen’s recommendations, where the small, medium, and large effect sizes were *d* ≥ 0.20, 0.50, and 0.80, respectively (Bonnert et al., 2019). A significance value of *p* < 0.05 indicated that the difference was statistically significant, while *P* < 0.01 indicated that the difference was very statistically significant.

## Results

**FIGURE 1:**
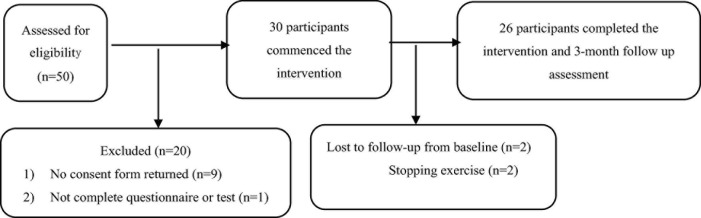
Consolidated standards of reporting trials flowchart.

A total of 30 children with obesity who met the inclusion criteria were included in this study. During the trial, 4 children withdrew and the data of 26 children with obesity (boys: 10; girls: 16) who completed the entire study were included in the final statistical analysis (Figure 1). The results of descriptive statistical analysis are shown in Table 1, including the height, weight, waist circumference, and hip circumference.

**TABLE 1:** Participant baseline demographic characteristics.

Demographic characteristics (*N* = 26)		
	Mean (±SD)	Frequencies (Counts)
Age (years)	10.15 (0.82)	
**Gender**		
Boys		10
Girls		16
Weight (kg)	59.69(11.24)	
Height (cm)	145.21(9.83)	
Waist(cm)	75.06(8.90)	
Hip (cm)	84.47(6.97)	

In this study, a total of 50 children met the inclusion criteria, 30 of which their parents agreed to allow their child to participate (i.e., 60% recruitment rate), and finally, the data of 26 children with obesity who completed the 8-week intervention and 3-month follow-up evaluation were included in the data analysis (Figure 1). During the online combination exercise intervention, only 4 obese children withdrew, and the remaining 26 children with obesity completed 3 months of follow-up tracking, with A retention rate of 86.7%. In addition, no adverse events were reported during the entire exercise intervention and follow-up. On average, obese children completed 90.6% of courses (29/32 courses) and completed 78.1% of courses (25/32 courses) and above.

As can be seen from Table 2, after 8 weeks of the online combination exercise intervention, the change in body fat percentage of children with obesity was statistically significant (*MD* = –3.126, *p* < 0.001, Cohen’s *d* = –1.777), but there was no significant difference in body fat percentage from intervention to the end of follow-up (*p* > 0.05), suggesting that the change from baseline to follow-up was persistent. BMI (*MD* = –0.175, *p* > 0.05, Cohen’s *d* = –0.256) and waist-to-hip ratio (*MD* = –0.009, *p* > 0.05, Cohen’s *d* = –0.097) after 8 weeks of online combination exercise intervention were not statistically significant (Table 2).

**TABLE 2:** Baseline, 8 weeks and 3 months obesity and mental health variable descriptive statistics.

Outcome	Observed mean (SD)			Baseline-8 weeks		8 weeks-3 months	
	Baseline	8 weeks	3 months	Mean change	Cohen’s d (95% CI)	Mean change	Cohen’s d (95% CI)
**Obesity**							
BMI	23.31(2.75)	23.14(2.63)	23.26(2.53)	–0.175	–0.256(–0.645,0.137)	0.118	0.030(–0.355,0.414)
Waist-to-hip ratio	0.887(0.05)	0.879(0.06)	0.874(0.05)	–0.009	–0.097(–0.482,0.289)	–0.004	–0.110(–0.494,0.277)
Bodyfat percentage	23.11(4.42)	19.98(3.65)	20.62(3.44)	−3.126**	–1.777(–2.392, –1.147)	0.646	0.157(–0.231,0.542)
**Mental health**							
Cognition	17.92(3.57)	18.577(3.23)	20.04(2.24)	0.654	0.212(–0.179,0.599)	1.462	0.600(0.176,1.013)
Thinking	16.73(3.90)	18.39(3.55)	19.42(2.40)	1.654*	0.603(0.179,1.017)	1.038	0.299(–0.097,0.690)
Personality	22.77(3.68)	22.12(3.64)	22.65(3.89)	–0.654	–0.137(–0.522,0.250)	0.538	0.150(–0.238,0.536)
Emotions	11.27(1.97)	11.73(2.03)	11.96(2.34)	0.462	0.207(–0.184,0.593)	0.231	0.095(–0.291,0.480)
Volitional behavior	19.42(2.63)	19.27(2.99)	20.19(2.37)	–0.154	–0.047(–0.431,0.339)	0.923	0.293(–0.102,0.683)

Outcomes are n, mean (SD), or as otherwise indicated. * *p* < 0.05 and ** *p* < 0.01.

After 8 weeks of the online combination exercise intervention, children with obesity had statistically significant changes in the thinking dimension scores (*MD* = 1.654, *p* < 0.001, Cohen’s *d* = 0.603), yet there was no significant difference in thinking dimension scores from 8 weeks after the intervention to the end of follow-up (*p* > 0.05), indicating that the change from baseline to follow-up was continuous. However, the cognitive dimension (*MD* = 0.654, *p*>0.05, Cohen’s *d* = 0.212), personality dimension (*MD* = –0.654, *p* > 0.05, Cohen’s *d* = –0.137), emotional dimension (*MD* = 0.462, *p*>0.05, Cohen’s *d* = 0.207) and volitional behavior (*MD* = –0.154, *p* > 0.05, Cohen’s *d* = –0.047) scores did not change significantly (Table 2).

As shown in Table 3, after 8 weeks of the online combination exercise intervention, the changes of total quality of life score (*MD* = 6.385, *p* < 0.05, Cohen’s *d* = 0.610) and the work attitude dimension score (*MD* = 1.346, *p* < 0.001, Cohen’s *d* = 0.744) were statistically significant. Moreover, there was no significant difference from the intervention to the end of follow-up (*p* > 0.05), indicating that the change from baseline to follow-up was continuous. However, there was no significant change in the scores of other dimensions of quality of life after online combination exercise intervention (Table 3).

**TABLE 3:** Baseline, 8 weeks and 3 months quality of life variable descriptive statistics.

Outcome				Baseline-8 weeks		8 weeks-3 months	
	Baseline	8 weeks	3 months	Mean change	Cohen’s d (95% CI)	Mean change	Cohen’s *d* (95% CI)
TSR	17.46(2.50)	17.27(2.93)	17.46(3.02)	–0.192	–0.065(–0.449,0.321)	0.192	0.058(–0.327,0.442)
PR	16.69(3.12)	17.65(2.06)	17.15(3.02)	0.962	0.469(0.059,0.871)	–0.500	–0.178(–0.564,0.211)
PCR	13.54(1.79)	13.15(2.52)	14.04(2.13)	–0.385	–0.153(–0.538,0.235)	0.885	0.299(–0.097,0.689)
LA	9.50(1.61)	9.85(1.74)	9.96(1.66)	0.346	0.183(–0.207,0.568)	0.115	0.066(–0.320,0.450)
Self-concept	11.96(2.41)	12.00(2.67)	11.54(2.90)	0.038	0.016(–0.368,0.400)	–0.462	–0.136(–0.521,0.252)
SS	15.50(2.70)	16.27(3.38)	17.27(2.25)	0.769	0.205(–0.185,0.592)	1.000	0.334(–0.064,0.726)
NE	11.65(2.21)	12.50(2.12)	13.08(1.96)	0.846	0.508(0.094,0.913)	0.577	0.237(–0.155,0.624)
Work attitude	8.73(2.49)	10.08(1.87)	10.04(1.71)	1.346**	0.744(0.302,1.174)	–0.038	–0.021(–0.405,0.364)
LC	6.54(1.56)	6.77(1.70)	6.31(1.74)	0.231	0.146(–0.242,0.531)	–0.462	–0.297(–0.687,0.099)
AO	8.35(2.53)	8.96(2.46)	8.65(2.31)	0.615	0.357(–0.043,0.751)	–0.308	–0.119(–0.503,0.268)
Athletic ability	7.65(2.45)	8.31(2.33)	8.31(2.62)	0.654	0.309(-0.088,0.700)	0.000	0.000(–0.384,0.384)
Self-satisfaction	20.08(2.67)	20.85(2.34)	20.96(2.52)	0.769	0.342(–0.057,0.734)	0.115	0.043(–0.342,0.427)
Other	5.54(1.17)	5.92(1.26)	6.23(1.24)	0.385	0.350(–0.049,0.743)	0.308	0.283(–0.112,0.673)
Total	153.19(15.00)	159.58(17.54)	161.00(18.35)	6.385*	0.610(0.185,1.025)	1.423	0.089(–0.297,0.474)

Outcomes are n, mean (SD), or as otherwise indicated. BMI, Body Mass Index; SD, standard deviation; CI, confidence interval; TSR, teacher-student relationship; PR, peer relationship; PCR, parent-child relationship; LA, learning ability; SS, somatic sensation; NE, negative emotions; LC, life convenience; AO, activity opportunity. * p < 0.05 and ** p < 0.01.

## Discussion

This is the first study to explore the impact of an online combination exercise intervention on the physical and mental health of Chinese children with obesity. The results of this study yielded a recruitment rate of 60%, but after three months of follow-up the retention rate reached 86.7%. No adverse events were reported throughout the trial. On average, children participated in 90.6% of the courses (29/32) and all the children completed 78.1% of the courses (25/32 courses) and above, which may have important practical implications for percentage of fat loss, the thinking dimension of mental health, and quality of life. It is also worth noting that the benefits of 8 weeks of an online combination exercise intervention can continue until the end of 3 months of follow-up.

Previous studies have found that inadequate facilities and lack of transportation can reduce children’s physical activity levels (Ward et al., 2017). In such a case, an online intervention can reduce the interference of these factors. In addition, the lack of children with obesity participating in the intervention courses during the intervention process (attendance rate: 92.9%) may have been caused by the Internet and social media applications. We believe that the future attendance rate of this intervention would be higher with the development of the Internet. Moreover, no adverse events occurred during the entire study process. Therefore, a suitable online combination exercise may be safe for children with obesity. However, many parents currently did not have enough knowledge of childhood obesity and did not recognize that their children had overweight or obesity (Vos and Welsh, 2010). This may possibly account for our recruitment rate of 60% in this study. Therefore, the physical literacy in parents and children in China is worth promoting and it is necessary to encourage parents/guardians to be aware of childhood obesity.

Previous studies have shown that physical activity can be an effective strategy to improve the symptoms of overweight or obesity (Monteiro et al., 2015**;** Ring-Dimitriou et al., 2019). The findings of this study also show a significant improvement in body fat percentage and a continuous improvement from the baseline to the end of the 3 months follow-up. Even if the BMI and waist-to-hip ratio did not show significant improvement, there was a trend of improvement. As it is known, obesity leads to a variety of physical and mental health risks (e.g., type 2 diabetes mellitus, stress, depression, etc.) (Lau et al., 2015). The prevalence of metabolic syndrome in children will also increase with the severity of obesity, and children with overweight status also often display heart biomarkers for increased risk of vascular dysfunction (Weiss et al., 2004). Therefore, online combination exercise may not only improve the obesity status of children but also reduce their associated health risks. However, this study did not explore other risk factors, meaning that further investigation in this line of inquiry is needed to fill this void. In addition, previous meta-analyses are still controversial in terms of exploring different exercise modalities for curbing and preventing childhood obesity. Some studies have shown that a combination exercise intervention of aerobic and resistance exercise had the greatest effect on obesity (Kelley et al., 2017), while others have shown that aerobic exercise alone is the best exercise modality for preventing obesity (Kelley et al., 2019). These findings need to be further confirmed by high-quality randomized controlled trials (RCT) in the future.

The mental health problems of children and adolescents have become a major public health issue (Granrud et al., 2019) that seriously affects the physical and mental health of adults (Halfon et al., 2013). Previous studies have shown that physical activity can improve young children’s cognition (Xiong et al., 2019; Gao et al., 2019a) and that moderate to intense physical activity can improve mental health problems (Rodriguez-Ayllon et al., 2019). Physical activity can be used as an effective means for young children to prevent future mental health problems (Poulsen et al., 2016). Our results show that online combination exercise could significantly improve obese children’s mental health and that this improvement continued after 3 months of follow-up. Previous studies have explained that thinking styles may affect children’s self-esteem and that higher self-esteem may cause them to have satisfaction in life, which also helps to further improve mental health (Ojha and Singh, 2015); however, some studies have shown that there is no evidence to prove that physical activity is associated with better mental health or reducing the symptoms of mental health disorders (Bell et al., 2019). Therefore, the relationship between physical activity and mental health still needs to be further explored by future high-quality research. Additionally, the other dimensions of mental health in this study did not show a statistically significant improvement. We assume that this may be related to our short intervention time. High-quality research is still needed in the future.

Previous studies have shown that children with high levels of physical activity exhibit a higher quality of life and subjective well-being (Sigvartsen et al., 2016). Low levels of physical activity can even reduce the quality of life by 30% (Pavlova et al., 2016), and increasing physical activity can create the best conditions and possibilities for improving the quality of life of young children (Brodáni et al., 2015). This is consistent with our research, which shows that online combination exercise significantly improved the quality of life of children with obesity. In addition, we found that the benefit lasted for three months, meaning that the quality of life benefit for Chinese children with obesity who participated in the online combination exercise during the summer and winter holidays continued throughout the entire semester. Moreover, the participants’ work attitude dimension in our study was significantly improved, and this benefit can also last for at least three months. Related research has explained the importance of homework attitudes for children and adolescents. For example, cultivating healthy homework attitudes for children and adolescents is an effective way to improve children’s physique and reduce their consumption of unhealthy foods (Pavlova et al., 2016), and a good homework attitude can reduce sedentary time in teens with obesity (Duncan et al., 2011) and improve their quality of life (Yi et al., 2019). In addition, forming a good work attitude is an effective way to prevent obesity (Zhang et al., 2016). Then, we can improve parents’ awareness of children’s obesity, enhance their physical activity by increasing their children’s interest, and balance the conflict between study and physical activity. Remote sports guidance may be an effective supplement.

The advantages of this study lie in the following: (1) this study provides preliminary evidence for the effectiveness of an online combination exercise intervention in children with obesity; (2) it explores the long-term benefits of an online combination exercise intervention on children with obesity; (3) combination exercise draws on the American College of Sports Medicine (ACSM) exercise recommendations and guidelines to ensure the safety of the intervention; and (4) the retention rate reached 92.9% during the entire test, which further verifies the feasibility of this study. However, there are some limitations in this study. First, this study only recruited samples from the Heze area of Shandong Province, China, who are all Han ethnicity. The feasibility of the study results in other regions or for other ethnicities needs further investigation. Secondly, our study followed a one-group pre-test and post-test design without a control group, which may limit the generalizability of our preliminary findings. Thirdly, we did not assess physical activity and dietary impacts. Finally, we did not conduct a personalized online exercise prescription intervention and dietary follow-up, which may also influence the effectiveness of the exercise intervention.

## Conclusion

The results of this study offer important implications for clinicians, child educators, and parents. Specifically, this study has provided preliminary evidence regarding the effectiveness of the use of an online combination exercise intervention for children with obesity to improve their weight status, mental health, and quality of life, which may confer long-term health benefits throughout their lifespan. However, to address the limitations of this study, our findings still need to be further confirmed by future interventions using a more rigorous trial design (e.g., RCT) and the further assessment of other health indicators.

## Data Availability Statement

The original contributions presented in the study are included in the article/supplementary material, further inquiries can be directed to the corresponding author.

## Ethics Statement

This study was approved by the Ethics Committee of Shandong Normal University (20180511). Written informed consent to participate in this study was provided by the participants’ legal guardian/next of kin.

## Author Contributions

XD and PY: data curation and investigation. XY: funding acquisition and writing—original draft. XD, MDi, and DM: methodology. MDi and ZG: project administration. XY and XD: supervision. XY, MDi, and ZG: writing—review and editing. All authors have read and approved the manuscript.

## Conflict of Interest

The authors declare that the research was conducted in the absence of any commercial or financial relationships that could be construed as a potential conflict of interest.

## Publisher’s Note

All claims expressed in this article are solely those of the authors and do not necessarily represent those of their affiliated organizations, or those of the publisher, the editors and the reviewers. Any product that may be evaluated in this article, or claim that may be made by its manufacturer, is not guaranteed or endorsed by the publisher.

## Abbreviations

BMI: Body Mass Index
SD: standard deviation
CI: confidence interval
TSR: teacher-student relationship
PR: peer relationship
PCR: parent-child relationship
LA: learning ability
SS: somatic sensation
NE: negative emotions
LC: life convenience
AO: activity opportunity.
